# Observations on the Analogy between Ophthalmic and Other Diseases, Being the Substance of a Paper Read at King's College, London

**Published:** 1842-04

**Authors:** 


Art. XI.—*Observations on the Analogy between Ophthalmic and other Diseases, being the substance of a Paper read at King's College, London.* By Charles Vines, m.r.c.s., Surgeon to the Reading Dispensary.—*London*, 1841. 8vo, pp. 32.

It appears from the preface, that it was before the Medical Society of King's College, in January, 1836, this paper was originally read. It is very creditable to the author's talents while yet a student.

